# Colorimetric Determination of Dissolved Oxygen: Assessment of Methodological Influences on Iodine Spectra, Isosbestic Point, Precision and Accuracy

**DOI:** 10.1021/acsomega.4c09084

**Published:** 2024-11-11

**Authors:** Su-Cheng Pai, Cheng-Ho Li, Brandon M. Stephens

**Affiliations:** Institute of Oceanography, National Taiwan University, 1 Section 4 Roosevelt Road, Taipei 106319, Taiwan

## Abstract

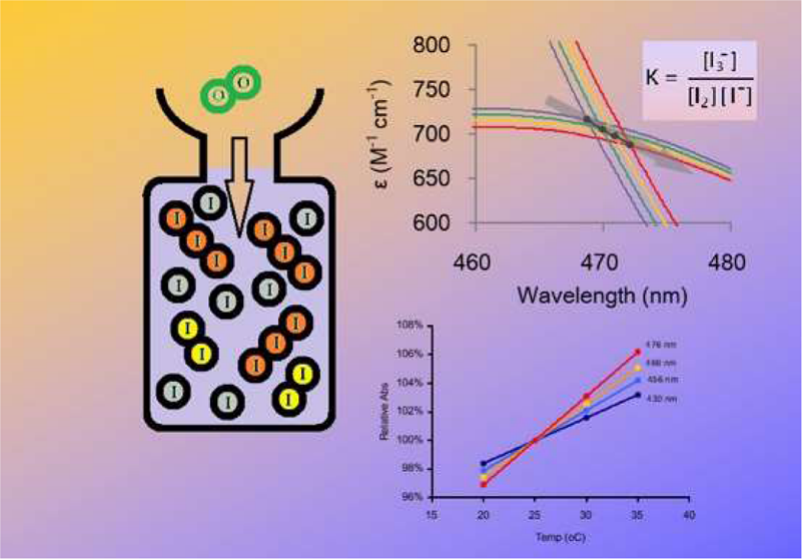

The colorimetric method for determining dissolved oxygen concentrations in freshwater and seawater samples essentially relies on measuring the absorbance of released I_2_ and I_3_^–^ in a mixed form. While this approach is relatively quick and convenient, it is susceptible to significant temperature effects during analysis, irrespective of field temperatures. Additionally, the influences of spectrophotometric absorbance wavelengths and iodine concentrations on oxygen concentration determinations are ambiguous. We found that, while iodine concentration and absorbance wavelength impart minor influences, temperature changes during sample analysis alter the fractionation of two major iodine species (I_2_ and I_3_^–^) and affect their spectra, with the latter effect imparting the greatest influence on oxygen concentrations. An empirical molar extinction coefficient was defined for the mixture (consisting of 96% I_3_^–^ and 4% I_2_), yielding values of 2282, 1115, and 792 M^–1^ cm^–1^ at 25 °C at the most commonly used wavelengths of 430, 456, and 466 nm, respectively. Altering the temperature led to variances in absorbance by 0.33%, 0.42%, and 0.51% °C^–1^ at 430, 456, and 466 nm, respectively. Therefore, for absorbances measured at 456 nm, a room temperature correction can be applied to the empirical molar coefficient by 1115 M^–1^ cm^–1^ × [1 + (room temperature – 25 °C) × 0.0042]. Reducing room temperature differences between samples and calibration solutions became the most important factor in maintaining the precision and accuracy of the measurement. With these precautions, a precision of ∼0.2% (coefficient of variation) over the normal surface water oxygen concentration of around 250 μM can be readily achieved at all tested wavelengths. This method offers a linear oxygen concentration range of 0–650 μM.

## 1 Introduction

The precise quantification of dissolved oxygen in freshwater and seawater is crucial to identifying subtle environmental differences in both temporal and spatial trends. Several methods are available to measure dissolved oxygen, including the Winkler titration,^1,2^ the colorimetric detection of iodine by spectrophotometer,^3−5^ and various oxygen sensors.^6,7^ Although oxygen sensors are becoming increasingly common in laboratory and field studies,^6^ data quality by this method still heavily relies on precise, accurate and reliable wet-chemical calibration. The classic Winkler titration is the first choice for this purpose. This method employs three analytical steps: oxygen fixation with a manganese hydroxide oxide precipitate, liberation of iodine by adding acid in the presence of excess iodide, and titration with thiosulfate. Since the titration process requires several hands-on steps, which can be time-consuming, several colorimetric methods^4,5,8^ have been proposed as alternatives to the titration procedure, especially for clean waters like open oceans and nonturbid freshwaters. The advantages of using colorimetric methods are not only due to their simplicity and high precision but also to the effectiveness of eliminating iodine vaporization loss during the measurement.

Although the colorimetric approach has been used as a routine tool for seagoing expeditions over the last three decades, several analytical concerns still need clarification.

(a) *Regarding reagent strengths*. Since the colorimetric method has been established for measuring a mixture, it is essentially empirical. It is assumed that the three species (i.e., I_2_, I_3_^–^, and I^–^) have reached equilibrium in the final solution. However, in reality, the oxygen within the sample and the added KIO_3_ both consume I^–^, thus the ratio of iodine species can be altered from one sample to another. The fractionation of iodine species must be calculated to identify these influences.
(b) *Regarding the wavelength chosen for detection*. A wavelength of 456 nm was first proposed by Pai et al.^4^ and then 430 nm by Roland et al.^5^ However, Labasque et al.^8^ suggested that the measurement should be carried out at an “isosbestic” wavelength of 466 nm where both I_2_ and I_3_^–^ species share the same molar extinction coefficient. According to this concept, the determination will not be affected if the iodine fraction is altered. As shown in Labasque et al.^8^ and as will be shown below, there was a significant temperature effect on the isosbestic point (the absorbance may change approximately 0.5% per 1 °C at 466 nm). The reason for this effect remains to be identified.
(c) *Regarding how to minimize the temperature effect*. Ocean water samples (i.e., as a depth profile) can be collected from in situ temperatures ranging between 2 and 35 °C. Additionally, adding sulfuric acid during the last step will suddenly release heat before detection. Analytical criteria need to be established to ensure all measurements (as well as the standardization) are made under the same conditions.

These concerns will be carefully examined in this study.

### 1.1 Equations Involved in the Colorimetric Winkler Reactions

While basic equations for the Winkler method are available in numerous literature sources,^4,5,8^ we present more detailed equations related to colorimetry, specifically, to focus on how the fractionation of iodine alters absorbance with increases in oxygen or iodate levels.

(i) Oxygen is fixed by adding Mn^2+^ and NaOH in a flared-mouth bottle, usually referred to as a biological oxygen demand (BOD) bottle, to form precipitates
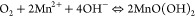
1
(ii) Iodine is released by dissolving the precipitates with acid in the presence of iodide
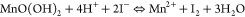
2
(iii) Iodine further reacts with excess iodide to form triiodide
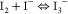
3
(iv) The final fractions of each iodine species are dependent upon the concentration of iodide present and the stability constant *K* for I_3_^–^
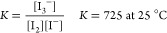
4According to Burger and Liebhafsky^9^ the *K* value at various temperatures *T* (°C) can be estimated by an empirical equation
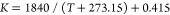
5
(v) The molar extinction coefficient ε_2_ for I_2_ can be obtained by preparing a known concentration of pure molecular iodine solution and measuring its absorbance
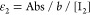
6where *b* is the cuvette light path (usually 1 cm).
(vi) The molar extinction coefficient ε_3_ for I_3_^–^ can be calculated by measuring the absorbance of a mixed solution of I_2_ and NaI and solving for the triiodide fraction. Assuming the final concentration of triiodide is *x*, *A* and *B* are the initial concentrations of I_2_ and I^–^, the equilibrium can be expressed as
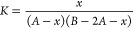
7where *B* > 2*A*. The final concentration of triiodide can be solved as
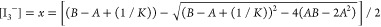
8And the final concentration of I_2_ is
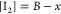
9The absorbance measured refers to a combination of two major iodine species
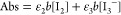
10Thus, the value of ε_3_ can be estimated following the equation below when ε_2_ is obtained previously
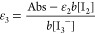
11
(vii) In a mixed I_2_ + I_3_^–^ solution, a molar ratio *r* can be defined as
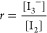
12The sum of I_2_ and I_3_^–^ gives total iodine ∑I_2_
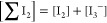
13If *r*, *b*, ε_2_, and ε_3_ are all fixed values, then eq 10 can be simplified as
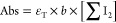
14where ε_T_ can be regarded as the “empirical molar extinction coefficient” for total iodine (i.e., the sum of I_2_ and I_3_^–^) at a given I^–^ concentration and a given temperature.
(viii) The calibration is usually made by adding a known amount of iodate to an iodide solution, which generates the brownish iodine color
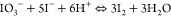
15In this reaction, each mole of iodate added will produce 3 mol of molecular iodine while consuming 5 mol of iodide, followed by a fraction of molecular iodine consuming another mole of iodide to form triiodide. Assuming the final concentration of triiodide is *x*, *C* and *B* are the initial concentrations of iodine and initial iodide (where *B* > 5*C*), the equilibrium can be expressed as
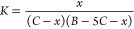
16Accordingly, the final concentration of triiodide will be
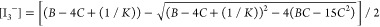
17The final concentration of molecular iodine is
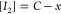
18Measuring the absorbance of this solution and calculating by eq 14 an ε_T_ value can be obtained in the spiking experiment
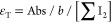
19
(ix) There are two types of reagent blanks involved in the colorimetric method, namely RB_1_ and RB_2_, representing the iodine impurities in the R2 reagent (NaI + NaOH) and oxygen contents in both R1 (Mn^2+^) and R2 reagents. The contributions of RB_1_ to the absorbance can be obtained experimentally by carrying out the same procedure for oxygen but adding reagents in reverse order (i.e., add R3 and R2 first, then R1). In our experience, the absorbance of RB_1_ is very small, mostly 0.000 and rarely exceeds 0.001.
  RB_2_ refers to the oxygen content within the dense R1 (3 M MnCl_2_ with a density of 1.30 g mL^–1^) and R2 (4 M NaI and 8 M NaOH with a density of 1.68 g mL^–1^) reagents. It is difficult to measure directly but can be evaluated according to Murray et al.^10^ who measured the oxygen concentrations in these two dense reagents. By calculating the amount of reagent used in a sample
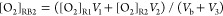
20where *V*_1_, *V*_2_, and *V*_3_ are added volumes for R1, R2, and R3. The excess [O_2_] for this reason is approximately 0.5 μM, which can be subtracted directly from each measured result.
(x) Converting measured absorbance to oxygen concentration: During the oxygen fixation step, for a bottle with an inner volume of *V*_b_, the actual volume of sample is *V*_b_ – *V*_1_ – *V*_2_ (e.g., 60 – 0.5 – 0.5 = 59 mL), and the final volume after adding R3 is *V*_b_ + *V*_3_ (e.g., 60 + 0.5 = 60.5 mL). Therefore, the resultant total iodine will be
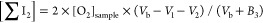
21By combining this equation with eq 13 and considering both reagent blanks, [O_2_]_sample_ = (Abs – RB1)/2 × (*V*_b_ + *V*_3_)/(*V*_b_ – *V*_1_ – *V*_2_) – [O_2_]_RB2_ or
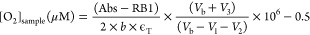
22The dilution term of (*V*_b_ + *V*_3_)/(*V*_b_ – *V*_1_ – *V*_2_) is nearly constant, even though the bottle volume *V*_b_ may vary significantly. For example, in the Shibala procedure,^4^ the value is 60.5/59 = 1.0254; in the Labasque^8^ procedure, it is 146.9/144.2 = 1.019. A bottle volume error of ±5% will only give a final variation of ±0.1%. Thus, for example, if ε_T_ is measured to be 1115 M^–1^ cm^–1^ at 25 °C using the Shibala procedure,^4^eq 22 can be simplified as
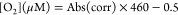
23

## 2 Experimental Section

### 2.1 Thermostat Spectrophotometer

**Figure 1:**
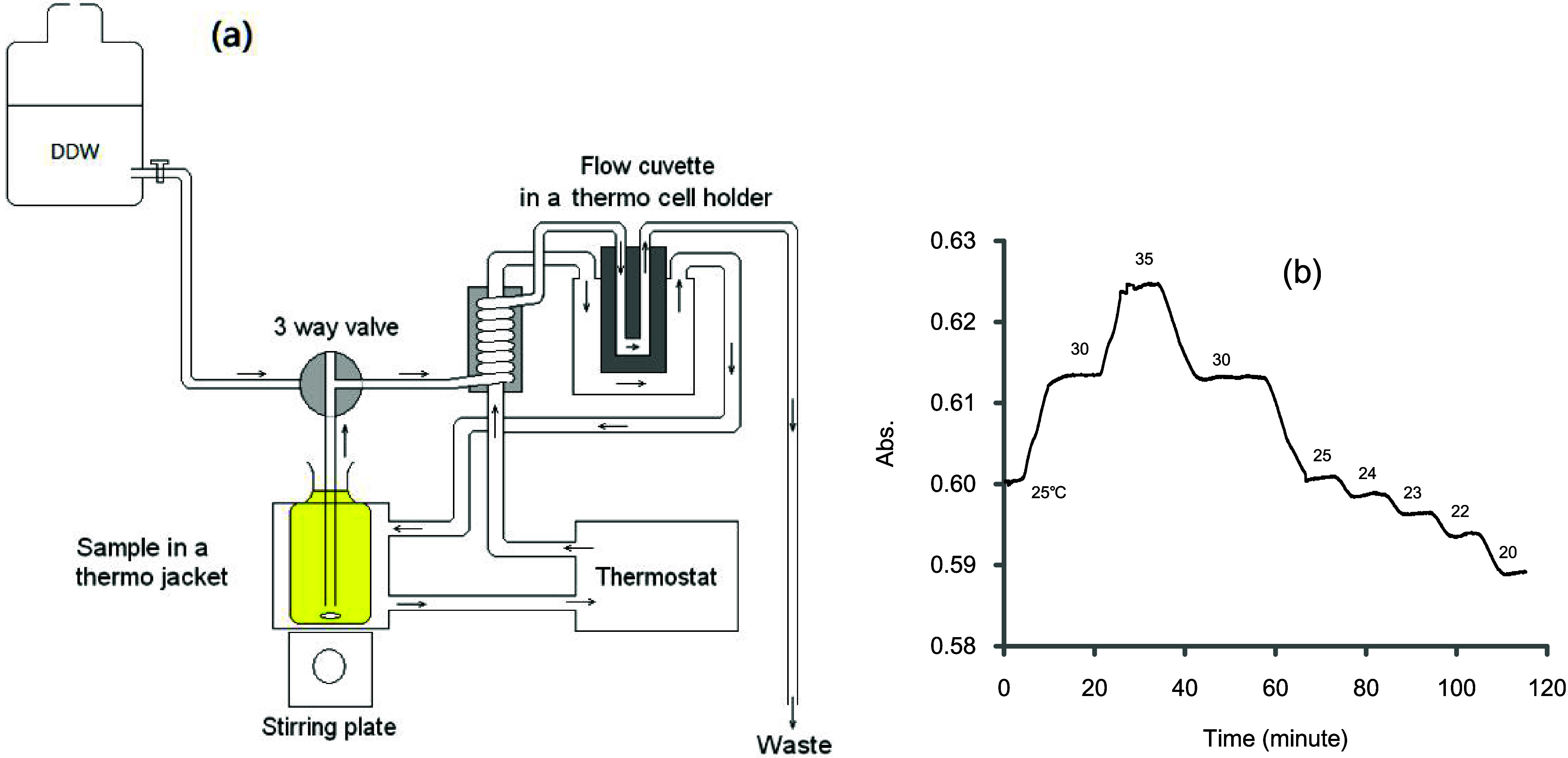
Layout of the water-circulation type thermostat spectrophotometer for the colorimetric Winkler method. (a) A thermo jacket outside the spectrophotometer holds a 60 mL BOD bottle, while a thermo cell holder inside the spectrophotometer contains a Hellma 1 cm dome-type flow cuvette. (b) The ramping rates for heating and cooling are roughly equal at 0.5 °C min^–1^. The efficiency of the water flow-circulating thermostat was demonstrated by filling the cuvette with an iodine solution and observing the variation of absorbance due to a change in the temperature setting. The absorbance indicates that when it becomes flat, the temperature has reached a steady state. The ramping rates for heating and cooling were similar at 0.5 °C min^–1^. If the temperature of the sample is different from the desired temperature, an appropriate waiting time is required before the scanning or detection operation.

Since the present study focuses on the temperature effect on the iodine color, most experiments were carried out under very precise temperature control. To do this, a double-beam spectrophotometer (Shimadzu 1800A, Japan) was equipped with a water-circulation type temperature-controlled flow system, including a sample-loading jacket and a cuvette holder (Figure 1a). The temperature of the water tank was controlled by a thermostat (Firstek, Taiwan) with a 1 KW heater and a 700 W cooler. A Hellma 1 cm dome-type flow cuvette (Hellma GmbH & Co, Germany) was used, which was surrounded by a thermo holder. The sample, contained within a 60 mL BOD bottle (Wheaton) was first placed in the thermo jacket and held for several minutes before being siphoned up to fill the cuvette volume. Then the sample was trapped in the cuvette to wait for another several minutes to reach a steady state before proceeding to either spectral scanning or to measure at a fixed wavelength. The ramping rate of the thermostat system was checked by an iodine color solution and found to be about 0.5 °C min^–1^ for both the heating and cooling processes (Figure 1b).

### 2.2 Procedure for Routine Measurement Using an Ordinary Spectrophotometer

**Figure 2:**
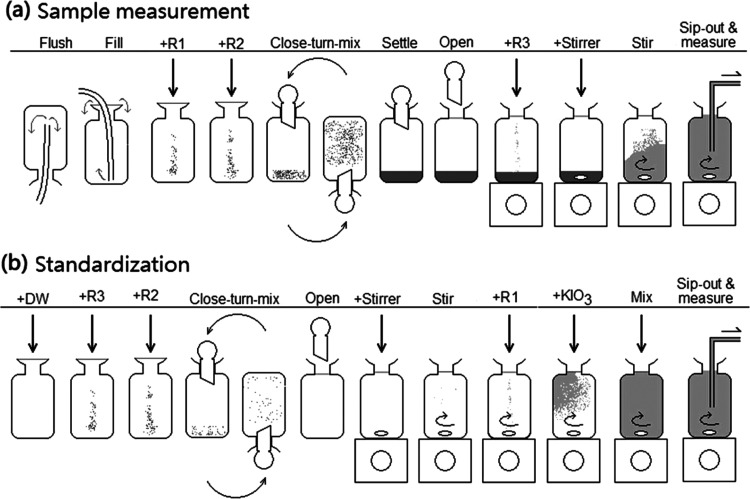
Illustration of the colorimetric Winkler method for oxygen measurement and calibration (following procedure outlined by Pai et al.^4^). Reagents used are R1 (containing 3 M Mn^2+^), R2 (containing 4 M NaI and 8 M NaOH) and R3 (10 N sulfuric acid). Potassium iodate (KIO_3_) is used as a standard. (a) Oxygen in a sample is fixed by adding R1 and R2 to form a precipitate. Upon measurement, the precipitate is dissolved by adding R3, releasing iodine and giving a brown color. The absorbance of the mixture is proportional to the [O_2_] in the original sample. (b) Distilled water (DW) is added with the same amounts of reagents but in reverse order. No precipitation is formed in the bottle, to which a known amount of KIO_3_ is added, which generates an iodine color. An empirical molar extinction coefficient for this mixture can be obtained for calibration purposes.

The routine procedure for measuring oxygen can be described graphically, as shown in Figure 2a. It is based on the protocol suggested by Pai et al.^4^ (also known as the Shibala procedure). For this, the water sample is filled into a BOD bottle (volume *V*_b_ = 60 mL), to which are added 0.5 mL of reagent R1 (containing 3 M MnCl_2_), 0.5 mL of reagent R2 (containing 8 M NaOH and 4 M NaI), the bottle is then stoppered, well mixed and allowed to stand for few hours until all precipitates are settled down to the bottom. Upon measurement, the bottle is opened and placed on a stir plate, and an aliquot of 0.5 mL of R3 (10 N sulfuric acid) is added while stirring. The brownish-colored solution is promptly sipped and transferred to fill a flow cuvette installed in a spectrophotometer, and the absorbance is recorded. The brownish color results from a mixture of molecular iodine (I_2_) and triiodide (I_3_^–^). Since their molar ratio is fixed, the absorbance is directly proportional to the oxygen concentration of the sample. DDW refers to double distilled water.

The procedure for the standardization is illustrated in Figure 2b. A BOD bottle filled with distilled water is treated with the same amounts of reagents but in reverse order. As the acidic R3 is added first, no precipitation will form; thus, the absorbance of this solution refers to the reagent blank, which has the same matrix as the oxygen measurement. The addition of known amounts of KIO_3_ to this solution produces an iodine color, and the absorbance obtained can be used to construct a calibration curve for the quantification of oxygen.

### 2.3 Definition of “Temperature”

In this study, two types of spectrophotometers were used. A thermostat spectrophotometer was employed to scan the spectra of pure I_2_ and I_3_^–^ solutions to identify the isosbestic point, while an ordinary spectrophotometer was used for routine measurements. To avoid any potential misunderstandings or confusion for readers, we here define the term “temperature” more clearly and precisely as follows:

*T*_b_: Temperature of the circulating water in the thermostat, which might slightly differ from the actual temperature of the flow cuvette if it is significantly higher or lower than room temperature.

*T*_r_: Room temperature in the laboratory where routine measurements are conducted.

*T*_w_: Temperature of the sample liquid after it has been left in the laboratory for at least 4 h to equilibrate with the surrounding environment. It is obtained by reading a thermometer placed in a bottle filled with water next to the spectrophotometer.

*T*_c_: Temperature of the sample immediately after acidification with R3 sulfuric acid, which releases heat and temporarily raises the temperature by roughly 0.8 °C.

*T*_a_: Actual temperature of the sample liquid trapped in the cuvette upon detection, which cannot be measured directly.

Since only *T*_w_ can be reliably recorded, in subsequent routine practice with an ordinary spectrophotometer, the term “temperature” should refer to *T*_w_.

## 3 Results and Discussion

### 3.1 Comparison of Existing Protocols

The two most adopted colorimetric protocols^4,8^ and two titration methods^11,12^ were compared based on bottle volumes, reagent strengths, and possible conditions that may cause variations in the final iodine fractionation. For simplicity, these four methods are referred to as Shibala, Labasque, APHA and Grasshoff in text and tables.

According to eq 4, the iodine fraction is heavily related to two factors, i.e., the I^–^ concentration and the formation constant K; the latter is a function of temperature. Theoretical calculations on alterations to the fractionation of iodine due to temperature are made and shown in Table 1. The original data provided by Burger and Liebhafsky^9^ showed a clear linear relationship between ln *K* and the reciprocal of temperature, leading to an empirical equation shown in eq 5. In this study, the *K* values were calculated to be 806, 725, 655, and 593 at temperatures of 20, 25, 30, and 35 °C, respectively. Four protocols were compared using those *K* values, assuming the oxygen concentration was 250 μM. For example, for the Shibala protocol an aliquot of 0.5 mL of R2 (containing 4 M I^–^) was added to a 60 mL bottle, resulting in an initial I^–^ concentration of 0.033 M in the final 60.5 mL solution. At 25 °C with a *K* value of 725, the final I_3_^–^ fraction is 95.82%, which will be reduced to 94.94% if the temperature is raised to 35 °C, as calculated according to eqs 8 and 9. The reducing trend is estimated to be −0.09% per 1 °C. The Labasque protocol uses the same R2 but adds 0.9 mL to a 146 mL bottle to give an initial I^–^ concentration of 0.0245 M in the final solution. At 25 °C the final I_3_^–^ fraction is 94.36% and will reduce to 93.49% at 35 °C. The decreasing trend is ca. −0.12% per 1 °C.

**Table 1:** Theoretical Calcultion of Iodine Fractionation Affected by Increasing Temperature Using Various Protocols^a^

						fractionation		
protocol	vol. of bottles (mL)	vol of R2 (mL)	[I^–^] in R2 (M)	final volume (mL)	temp (°C)	(I_3_^–^)%	(I_2_)%	change of (I_3_^–^)% per °C
Shibala	60	0.5	4	60.5	20	96.22%	3.78%	
					25	95.82%	4.18%	–0.09%
					30	95.39%	4.61%	
					35	94.94%	5.06%	
Labasque	146	0.9	4	146.9	20	94.89%	5.11%	
					25	94.36%	5.64%	–0.12%
					30	93.79%	6.21%	
					35	93.19%	6.81%	
APHA	300	1	0.87	301	20	56.60%	43.40%	
					25	54.17%	45.83%	–0.87%
					30	51.82%	48.18%	
					35	49.50%	50.50%	
Grasshoff	50	0.5	3.61	50.5	20	96.51%	3.49%	
					25	96.13%	3.87%	–0.08%
					30	95.74%	4.26%	
					35	95.32%	4.68%	

a Calculations were based on an assumed oxygen concentration of 250 μM.

For the APHA method, in which an aliquot of 1 mL of 0.87 M I^–^ is added to a 300 mL bottle, the starting I^–^ concentration is 0.0029 M. It does not provide enough [I^–^] to give a high I_3_^–^ fraction value. At 25 °C the I_3_^–^ fraction is 54.17% and drops to 49.5% at 35 °C with a decreasing trend of −0.87% per 1 °C. The increase of I_2_ fraction may induce vaporization loss,^13,14^ therefore it is not recommended for the colorimetric approach. In contrast, the Grasshoff protocol provides a more reasonable high I^–^ concentration (0.0357 M) and results in high I_3_^–^ fraction of 96.13% at 25 °C, similar to that of the two colorimetric protocols.

In general, raising the temperature will lead to a slight decrease in I_3_^–^ and an increase in I_2_. However, by using an I^–^ concentration of >0.03 M, these variations can be effectively limited to a negligible level within the normal oxygen concentration range.

### 3.2 Change of Iodine Fractionation Due to Oxygen Consumption

When oxygen is fixed and converted to I_2_, it consumes I^–^ as indicated by eqs 2 and 7, thus causing a slight decrease in I^–^ concentration and changing the final I_2_ fractionation. A theoretical calculation is also made for the four protocols. The results are summarized in Table 2.

**Table 2:** Theoretical Calculation of Iodine Fractionation Affected by Increasing Oxygen Concentration Using Various Protocols^a^

						fractionation		
protocol	vol. of bottles (mL)	vol. of R2 (mL)	[I^–^] in R2 (M)	final volume (mL)	assumed [O_2_] (μM)	(I_3_^–^)%	(I_2_)%	change of (I_3_^–^)% per 100 μM O2
Shibala	60	0.5	4	60.5	100	95.93%	4.07%	
					200	95.86%	4.14%	–0.07%
					300	95.78%	4.22%	
Labasque	146	0.9	4	146.9	100	94.55%	5.45%	
					200	94.42%	5.58%	–0.14%
					300	94.29%	5.71%	
APHA	300	1	0.87	301	100	63.20%	36.80%	
					200	57.53%	42.47%	–11.14%
					300	50.38%	49.62%	
Grasshoff	50	0.5	3.61	50.5	100	96.23%	3.77%	
					200	96.17%	3.83%	–0.06%
					300	96.10%	3.90%	

a Calculations were made at a temperature of 25 °C.

For the Shibala procedure, the starting I^–^ concentration is 0.033 M within the bottle. Assuming the oxygen concentration of a sample is 100 μM, the final concentrations for I^–^, I_2_ and I_3_^–^ will be 32855, 7.95, and 187.1 μM, respectively, with a [I_3_^–^]/[I_2_] ratio of 23.55. This means that 95.93% of total iodine will be I_3_^–^ and 4.07% will be I_2_ in the final solution. Increasing the oxygen concentration from 100 to 300 μM, I_3_^–^ only drops slightly to 95.78%. The decreasing trend is ca. −0.07% per 100 μM O_2_.

For the Labasque procedure, at a dissolved oxygen concentration of 100 μM, the final concentrations of I^–^, I_2_ and I_3_^–^ will be 24299, 10.7, and 185.6 μM, with a molar ratio of 17.35 and a final I_3_^–^ of 94.55%. Increasing the oxygen concentration from 100 to 300 μM decreases the fraction slightly to 94.29%, and the decreasing trend is also very small, at −0.14% per 100 μM O_2_.

Calculations of the two titration protocols are also given in Table 1. It is clear that the APHA procedure uses a comparatively larger bottle but weaker reagent strength for I^–^, giving a very low [I_3_^–^]/[I_2_] ratio of 1.72 at an [O_2_] of 100 μM. The ratio drops further as [O_2_] increases, which indicates that the I_2_ fraction turns gradually to nearly 50% at an [O_2_] of 300 μM. The Grasshoff procedure uses a smaller bottle and a stronger I^–^ strength, giving a much higher [I_3_^–^]/[I_2_] ratio of 25.5 at an [O_2_] of 100 μM, this is comparable to the high ratios provided by the Shibala and Labasque procedures.

### 3.3 The Iodine Spectra

Although the absorbance measured by the colorimetric method refers to a mixture of I_2_ and I_3_^–^, it is possible to obtain their contribution individually by observing the spectra of a pure I_2_ standard solution and a nearly pure I_3_^–^ solution (following eqs 6–19). Two solutions were prepared for this purpose and they were scanned between 400 and 700 nm at 20, 25, 30, and 35 °C. The first solution contained 0.0563 g I_2_ flakes (purity 99.8%) in 1 L of water, giving an [I_2_] of 224 μM. The second solution contained 0.0527 g of I_2_ flakes and 35.54 g of NaI in 1 L giving a final [I^–^] of 0.237 M, a total [I_2_] of 207 μM, and a I_3_^–^ fraction of >99%.

**Figure 3:**
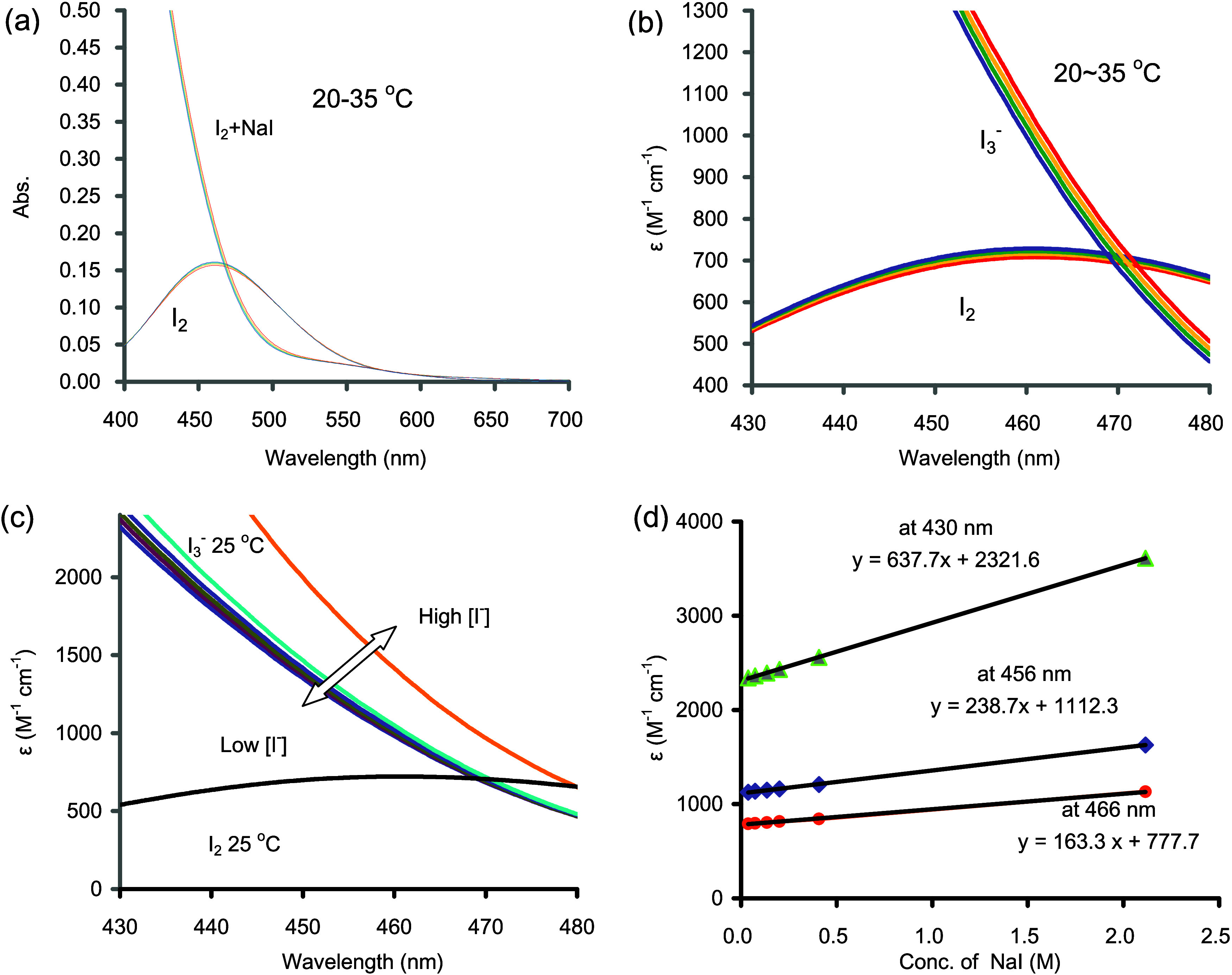
(a) Direct wavelength scan (400–700 nm) of two iodine solutions at various temperatures (*T*_b_ = 20–35 °C). The pure I_2_ solution containing 0.0563 g I_2_ flakes in 1 L gives an [I_2_] of 221 μM. The NaI+I_2_ solution is a mixture of 0.0527 g I_2_ flakes and 34.54 g NaI in 1 L. (b) Molar extinction coefficients (400–480 nm) for I_2_ and I_3_^–^ at different temperatures: (blue) 20 °C (green) 25 °C (yellow) 30 °C (red) 35 °C. (c) Molar extinction coefficients for I_2_ (no iodide) and I_3_^–^ (with different iodide concentrations). (d) The molar extinction coefficients for I_3_^–^ at 430, 456, and 466 nm show a linear correlation with the concentration of iodide. The intercept of the regression indicates a hypothetical value.

The absorption spectra of the two solutions are shown in Figure 3a. There are two major spectra for the pure I_2_ and the I_2_+NaI mixture, each consisting of 4 lines revealing the spectra at 4 different temperatures. The two bunches of spectra have two crosses, matching the results reported by Morel^15^ and Labasque et al.,^8^ who described that the two species have identical absorptivities at 466 and 580 nm, respectively.

The influence of temperature on the absorption spectra was statistically significant, but the trends were opposite to that of the fractionation of iodine species. In Figure 3b, the absorbance scale was converted to molar extinction coefficient scale. For the pure I_2_ solution, the highest absorption occurred at 460 nm. The molar extinction coefficient (ε_2_) measured at 20 °C was higher than that at 35 °C, with values of 729 and 708 M^–1^cm^–1^, respectively (Table 3). The decreasing trend was −0.15% °C^–1^. For the predominantly I_3_^–^ solution, the spectrum appeared to shift up or to the right as temperature increased. At 456 nm, the molar extinction coefficient (ε_3_) was 1145 M^–1^ cm^–1^ at 20 °C and 1222 M^–1^ cm^–1^ at 35 °C (Table 3), with an increasing trend of 0.44% °C^–1^.

**Table 3:** Effect of Temperature on the Molar Extinction Coefficients

	temperature				
wavelength (nm)	20 °C	25 °C	30 °C	35 °C	effect of temp. (1/°C)
Molar Extinction Coeffient for I_2_ (ε_2_, M^–1^cm^–1^)^a^					
430	543	540	536	531	–0.15%
456	724	717	711	703	–0.19%
460	729	722	715	708	–0.19%
466	723	717	711	704	–0.18%
469	715	709	704	697	–0.17%
470	712	706	700	694	–0.17%
476	685	680	676	670	–0.15%
Molar Extinction Coefficient for I_3_^–^ (ε_3_, M^–1^cm^–1^)^b^					
430	2444	2484	2525	2569	0.34%
456	1145	1171	1195	1222	0.44%
460	997	1022	1045	1070	0.48%
466	797	819	840	863	0.53%
469	709	730	749	771	0.57%
470	682	703	722	742	0.58%
476	538	556	573	592	0.64%

a A solution containing 0.0563 g iodine flake in 1 L.

b A solution containing 0.527g I_2_ flake and 35.542 g NaI in 1 L.

### 3.4 The Effect of Iodide Concentration

In this experiment, six solutions were prepared by transferring different amounts of NaI (i.e., 0.571, 1.103, 2.027, 3.025, 6.116, and 31.711 g) (mol. wt. = 149.89) with distilled water into a 100 mL flask. To each flask, 1 mL of 4972 μM KIO_3_ solution and 0.1 mL of 10 N sulfuric acid were added, and the final volume was adjusted to the mark. These solutions contained starting concentrations of 0.038, 0.074, 0.135, 0.202, 0.408, and 2.116 M of I^–^. These solutions were loaded into the flow cuvette at a fixed temperature of 25 °C for scanning between 430 and 480 nm. The results are shown in Figure 3c and Table 4.

**Table 4:** Effect of I^–^ Concentration to the Molar Extiction Coefficient of I_3_^–,^^a^,^b^

Experimental Condition						
[I^–^](M)	0.038	0.074	0.135	0.202	0.408	2.116
[I_3_^–^](μM)	143.8	146.4	147.6	148.1	148.7	149.1
[I_2_](μM)	5.34	2.78	1.52	1.02	0.50	0.10
[I_3_^–^]/[I_2_]	27	53	97	146	295	1533
(I_3_^–^)%	96.42%	98.14%	98.98%	99.32%	99.66%	99.93%

Apparent Extinction Coefficient for I_3_^–^ (M^–1^ cm^–1^)						
ε_3_ (430 nm)	2376	2396	2427	2467	2599	3672
ε_3_ (456 nm)	1127	1136	1147	1163	1208	1629
ε_3_ (466 nm)	791	797	805	815	842	1131

Apparent Extinction Coefficient for ∑I_2_ (M^–1^ cm^–1^)						
ε_T_ (430 nm)	2310	2362	2407	2454	2592	3670
ε_T_ (456 nm)	1112	1128	1143	1160	1207	1628
ε_T_ (466 nm)	788	796	804	814	842	1130

a Each NaI solution was added with 1 mL of 0.4972 mM KIO_3_.

b Experiments were carried out at 25 °C.

Under extremely high concentrations of I^–^ (e.g., >0.2 M), the I_3_^–^ fraction exceeds 99% (Table 4), but the absorbance still increases with I^–^ concentration. This phenomenon could be attributed a further complexation of I_3_^–^ to (I_5_^–^).^16^ However, in practical applications, I^–^ concentrations typically do not exceed 0.04 M, rendering the formation of I_5_^–^ negligible.

### 3.5 The Isosbestic Point

Labasque et al.^8^ suggested that the colorimetric measurements for [O_2_] should be made at an isosbestic point of 466 nm, at which the molar extinction coefficients for both I_2_ and I_3_^–^ species are identical. However, the isosbestic point was found to be around 470 nm in the current study. Experimental results have demonstrated that the isosbestic point shifts toward higher wavelengths and lower intensities by increasing temperature or [I^–^].

For instance, at constant [I^–^], the isosbestic point (λ_iso_) was found to be 468.7 nm at 20 °C, but drifted to 472.1 nm as the temperature increased to 35 °C (Figure 3b and Table 5). The corresponding molar extinction coefficient (ε_iso_) dropped from 716.5 M^–1^ cm^–1^ at 20 °C and to 687.6 M^–1^ cm^–1^ at 35 °C.

**Table 5:** Isosbestic Point Affected by Temperature and I^–^ Concentration

*T* (°C)	[I^–^] (M)	λ(iso) (nm)	ε(iso) (M^–1^ cm^–1^)
Change Temperature			
20	0.237	468.7	716.5
25	0.237	469.9	705.9
30	0.237	470.9	698.1
35	0.237	472.1	687.6
Change [I^–^]			
25	0.038	468.6	710.2
25	0.074	468.9	709.4
25	0.135	469.2	708.0
25	0.202	469.6	706.9
25	0.408	470.5	704.2
25	2.116	479.8	657.4

Iodide concentrations can also influence the isosbestic point. Under a fixed temperature of 25 °C, the isosbestic point drifted in a similar way as described above. At an [I^–^] of 0.038 M, the isosbestic wavelength was 468.6 nm and drifted to a higher wavelength as concentrations increased (Figure 3c and Table 5). At an extremely high [I^–^] of 2.116 M, the isosbestic wavelength (λ_iso_) was found to be 479.8 nm. The isosbestic molar extinction coefficient (ε_iso_) dropped from 710.2 to 657.4 M^–1^ cm^–1^.

### 3.6 Effect of Ionic Strength

For samples with high ionic strength, such as seawater, there is a concern that the activity coefficients of different species might change, potentially altering the formation constant *K*. However, previous studies have shown that the iodine spectra are not affected by salinity between wavelengths of 430 and 470 nm.^4^ This may be explained according to eq 4, given that both I^–^ and I_3_^–^ are monovalent ions and are present in the numerator and denominator, their activity coefficients tend to cancel each other out. This leaves only a small variation due to the activity of the nonvalent I_2_ species. In an I_3_^–^-dominating mixed solution, such variation is very small and can be reasonably ignored.

### 3.7 Oxygen Measurement and Calibration of ε_T_ by Spiking with KIO_3_

The calibration of ε_T_ was made by spiking various amounts of KIO_3_ standard solution to a distilled water sample and adding all the reagents in reverse order. The bottle was placed on a stir plate, and known amounts of KIO_3_ were added while stirring (e.g., 1.00–6.50 mL of 5000 μM KIO_3_). The absorbances recorded (at 456 nm in this case) were used to calculate the empirical molar extinction coefficient ε_T_ following eqs 19 and 20. Results are shown in Table 6. Basically, the empirical molar extinction coefficients were very consistent in the lower concentration range (0–1000 μM ∑I_2_), to be 1115 ± 3 M^–1^ cm^–1^ at *T*_w_ = 25 °C. The upper linear threshold of the calibration curve was defined as the concentration at which the slope value is dropped by 1% comparing to that at low concentration range. In the present case, the linear range was 0–1300 μM ∑I_2_, equivalent to an oxygen concentration range of 0–650 μM.

**Table 6:** Standardization of the Colorimetric Protocol by Spiking Known Amounts of Potassium Iodate at *T*_w_ = 25 °C^a^

*V*_b_ (mL)	spiked *V* (mL)	final V (mL)	[∑I_2_] final (μM)	abs (456 nm)	ε_T_ (M^–1^ cm^–1^)	(I_3_^–^) %	(I_2_) %
60.02	0.00	60.52	0.0	0.001			
60.47	0.00	60.97	0.0	0.001			
60.71	0.00	61.21	0.0	0.001			
60.31	1.00	61.81	242.0	0.271	1116	95.7%	4.3%
59.68	1.00	61.18	244.4	0.274	1117	95.8%	4.2%
61.07	1.00	62.57	239.0	0.268	1117	95.7%	4.3%
60.75	1.50	62.75	357.5	0.400	1116	95.6%	4.4%
60.12	1.50	62.12	361.1	0.405	1119	95.6%	4.4%
60.32	1.50	62.32	360.0	0.403	1117	95.6%	4.4%
60.34	2.50	63.34	590.3	0.660	1116	95.3%	4.7%
59.77	2.50	62.77	595.6	0.665	1115	95.4%	4.6%
62.02	2.50	65.02	575.0	0.643	1116	95.2%	4.8%
61.09	3.00	64.59	694.6	0.775	1114	95.1%	4.9%
60.00	3.00	63.50	706.5	0.790	1117	95.2%	4.8%
59.96	3.00	63.46	707.0	0.791	1117	95.2%	4.8%
59.48	3.50	63.48	824.6	0.918	1112	95.1%	4.9%
59.93	3.50	63.93	818.7	0.914	1115	95.0%	5.0%
59.90	3.50	63.90	819.1	0.912	1112	95.0%	5.0%
60.98	4.00	65.48	913.6	1.018	1113	94.8%	5.2%
59.37	4.00	63.87	936.6	1.043	1113	94.9%	5.1%
61.76	4.00	66.26	902.8	1.005	1112	94.7%	5.3%
59.46	4.50	64.46	1044.0	1.160	1110	94.7%	5.3%
60.64	4.50	65.64	1025.3	1.142	1113	94.6%	5.4%
61.44	4.50	66.44	1012.9	1.126	1111	94.6%	5.4%
59.93	5.00	65.43	1142.8	1.265	1106	94.5%	5.5%
59.81	5.00	65.31	1144.9	1.267	1106	94.5%	5.5%
61.22	5.00	66.72	1120.7	1.240	1106	94.4%	5.6%
59.36	5.50	65.36	1258.5	1.390	1104	94.4%	5.6%
60.67	5.50	66.67	1233.7	1.367	1107	94.3%	5.7%
60.25	5.50	66.25	1241.5	1.375	1107	94.3%	5.7%
61.39	6.00	67.89	1321.7	1.460	1104	94.0%	6.0%
59.61	6.00	66.11	1357.3	1.500	1104	94.1%	5.9%
59.42	6.00	65.92	1361.2	1.508	1107	94.2%	5.8%
59.30	6.50	66.30	1466.2	1.616	1102	94.0%	6.0%
59.89	6.50	66.89	1453.2	1.604	1103	93.9%	6.1%
59.97	6.50	66.97	1451.5	1.602	1103	93.9%	6.1%

a A stock solution of 5.00 mM KIO_3_ was used for spiking.

### 3.8 Temperature Variation in Field Application

**Figure 4:**
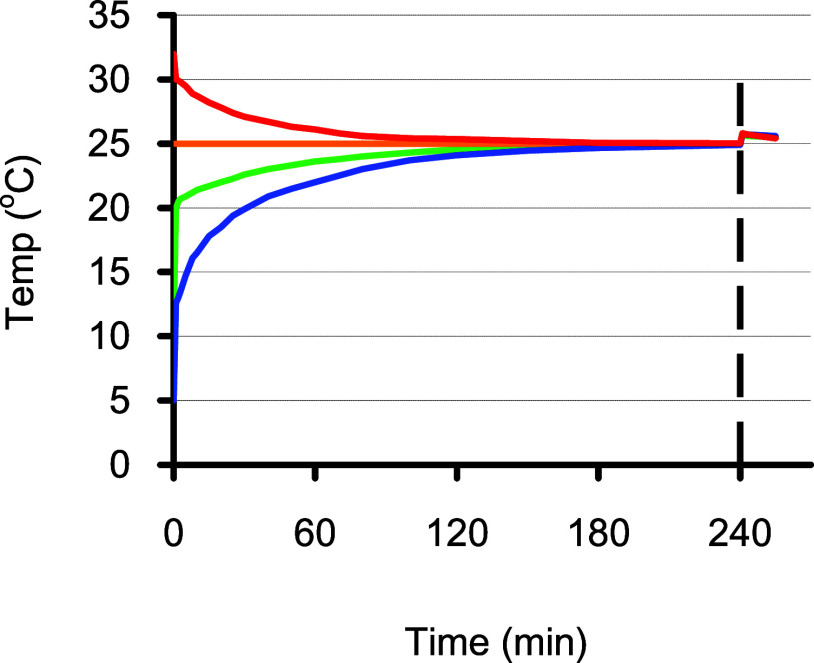
Changes of temperature in sampling operation. Four water samples with initial temperatures of 5, 10, 25, and 32 °C were filled into BOD bottles following standard procedures,^4^ and the temperatures were monitored for 240 min. The maximum difference was 4.1 °C at 60 min, but narrowed down to 1.2, 0.5, and 0.2 °C after 2, 3, and 4 h. After the addition of 0.5 mL of 10 N sulfuric acid at 4 h, the temperature in each bottle was raised by ca. 0.8 °C.

During open ocean field work, samples are often from different depths, which may cover a wide temperature range. A test was made to quantify the equilibration time required to minimize the temperature effect in a batch of environmental samples with different temperatures. To do this, aliquots of distilled water were adjusted to 32, 25, 10, and 5 °C, filled into the bottles, and then reagents were added. The temperatures inside those bottles were monitored for 4 h. After 1 h, the maximum temperature difference among the four bottles was reduced to 4.1 °C (Figure 4). The gap was further narrowed to 1.2 °C after 2 h, then 0.5 °C after 3 h, and 0.2 °C after 4 h. Upon adding the sulfuric acid, the temperature of each bottle was suddenly raised by 0.8 °C, and then gradually cooled down to the room temperature of nearly 25 °C (Figure 4). If the final temperature difference can be limited to less than 0.2 °C, the temperature influence on sensitivity (0.42% °C^–1^) can be reasonably controlled to less than 0.1%.

Although a thermostat spectrophotometer is always preferable, most laboratories are not equipped with such thermostat devices. It should also be noted that the temperature in the cuvette chamber is typically warmer than outside the instrument. If the laboratory environment is 25 °C, the actual detection temperature may be higher by 1–2 °C. Consequently, the empirical molar extinction coefficient obtained may be raised by ca. 1%. Analysts may not need to know the exact temperature during detection but can ensure all samples (including the calibration samples) undergo the same process. In this way, a highly precise measurement can still be expected without using a thermostat.

### 3.9 Precision and Accuracy for Routine Analysis

A replicate test was carried out on an air-saturated distilled water sample pickled at 22.0 °C and measured at *T*_w_ = 24.8 °C, the resultant absorbances (*n* = 6) were 1.097 ± 0.002, 0.536 ± 0.001 and 0.381 ± 0.001 at wavelengths of 430, 456, and 466 nm, respectively, with a coefficient of variation (c.v.) of ∼0.2% at normal oxygen saturation level. It should be noted again that the variation in bottle volume does not contribute more than 0.1% to the variation in oxygen concentration, as described in eq 22.

The accuracy of this colorimetric method depends solely on the spiking with known amounts of KIO_3_, deriving an empirical molar extinction coefficient ϵ_T_. Before analyzing each batch of samples, calibration must be performed carefully under a constant temperature *T*_w_. In our laboratory, after a decade of experience, we have consistently obtained an ϵ_T_ value of 1115 ± 5 M^–1^ cm^–1^ at 456 nm when *T*_w_ = 25 ± 0.5 °C. If the laboratory temperature deviates from 25 °C, a modified empirical equation can be used
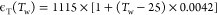
24This equation is valid under the conditions where 20 °C < *T*_w_ < 30 °C and 0.000 < Abs < 1.400.

## 4 Conclusions

The colorimetric Winkler method indeed offers advantages in the amount of time required and simplicity over traditional titration methods for determining dissolved oxygen concentration in water samples. Three factors have been identified to have influences on the precision and accuracy. These include:

(i) *Alteration of the fractionation due to change of temperature on the formation constant K.* In the Shibala procedure,^4^ the magnitudes are −0.09% °C^–1^ for I_3_^–^ and +2.05% °C^–1^ for I_2_. In the Labasque^8^ procedure, the magnitudes are −0.12% °C^–1^ for I_3_^–^ and +2.02% °C^–1^ for I_2_.
(ii) *Consumption of I*^–^*by oxygen leads to a decrease in the I*_3_^–^*but an increase in the I*_2_. The change of fractions in I_3_^–^ and I_2_ for the Shibala procedure are −0.07% and +1.73% per 100 μM O_2_ change, respectively. Meanwhile, for the Labasque procedure, the changes are −0.14% and +2.34% per 100 μM O_2_ change, respectively.
(iii) *Changes in the absorptivities of both iodine species due to temperature effects.* At 456 nm, the molar extinction coefficient increases by approximately +0.44% °C^–1^ for I_3_^–^, decreasing by −0.19% °C^–1^ for I_2_. At 466 nm, the trends are comparable, with an increase of +0.53% °C^–1^ for I_3_^–^ and a decrease of −0.18% °C^–1^ for I_2_.

**Figure 5:**
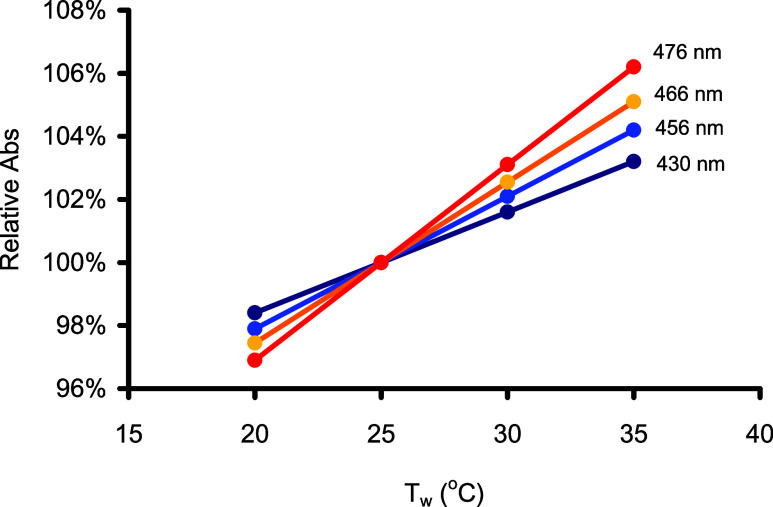
Overall temperature effect on the sensitivity at various wavelengths (430, 456, 466, and 476 nm) demonstrated as relative absorbance (w.r.t. *T*_w_ = 25 °C). Slopes are 0.33, 0.42, 0.51, and 0.62% per 1 °C at the corresponding wavelengths, respectively.

The combined result, as seen in absorbance, indicates an apparent temperature effect of +0.33%, +0.42%, and +0.51% °C^–1^ at wavelengths of 430, 456, and 466 nm, respectively. Figure 5 illustrates this effect by comparing absorbances measured at different room temperatures from those measured at 25 °C.

Based on the above findings, temperature control emerges as the most critical factor influencing the sensitivity, precision, and accuracy of the colorimetric method. While the temperature effect may be unavoidable, it can be minimized by ensuring a sufficiently high [I^–^] (>0.03 M) and allowing all samples, as well as standard solutions, to equilibrate with the laboratory environment for at least 4 h. By implementing these conditions, the impact of temperature fluctuations on the colorimetric method can be effectively mitigated, thus enhancing the reliability of the [O_2_] measurements.

Given that variations caused by iodine fractionation are no longer significant, it is reasonable to conclude that detection does not necessarily need to be at the isosbestic wavelength; a wide range from 430 to 476 nm can be selected for detection, providing flexibility in choosing the most suitable wavelength for different purposes.

Considering the naturally occurring [O_2_] range of 100–800 μM in most environmental applications, the sensitivity of detecting at 430 nm would be too high (A430/A456 = 2.05), as it could potentially exceed the linearity range in absorbance. Conversely, detecting at 466 nm might lose significant sensitivity (A466/A456 = 0.71) without necessarily improving precision. Therefore, detection made at 456 nm, as suggested by the Shibala procedure,^4^ offers a balanced compromise between sensitivity and linearity, as well as accounting for the influence of the temperature effects.

This work provides valuable analytical guidance for achieving very precise measurements of oxygen concentration using colorimetry. However, it is worth noting that the current application is still limited to oligotrophic waters, such as nonturbid freshwaters and open oceans. Corrections can be applied to turbid waters by adopting the procedures suggested by Roland et al.^5^ Further research will be continued to evaluate the potential of expanding the Shibala^4^ method to correct for turbidity and a suite of other interferences including nitrite, hydrogen peroxide, and iodate.
